# Supplementary material for: Role of TRPC3 in Right Ventricular Dilatation under Chronic Intermittent Hypoxia in 129/SvEv Mice
Source: Int J Mol Sci. 2023 Jul 10;24(14):11284. doi: 10.3390/ijms241411284 (PMC10379021; doi:10.3390/ijms241411284)
Supplement: Supplementary file 1 [file ijms-24-11284-s001.zip › ijms-2468448-supplementary.pdf]

Supplementary Figure S1

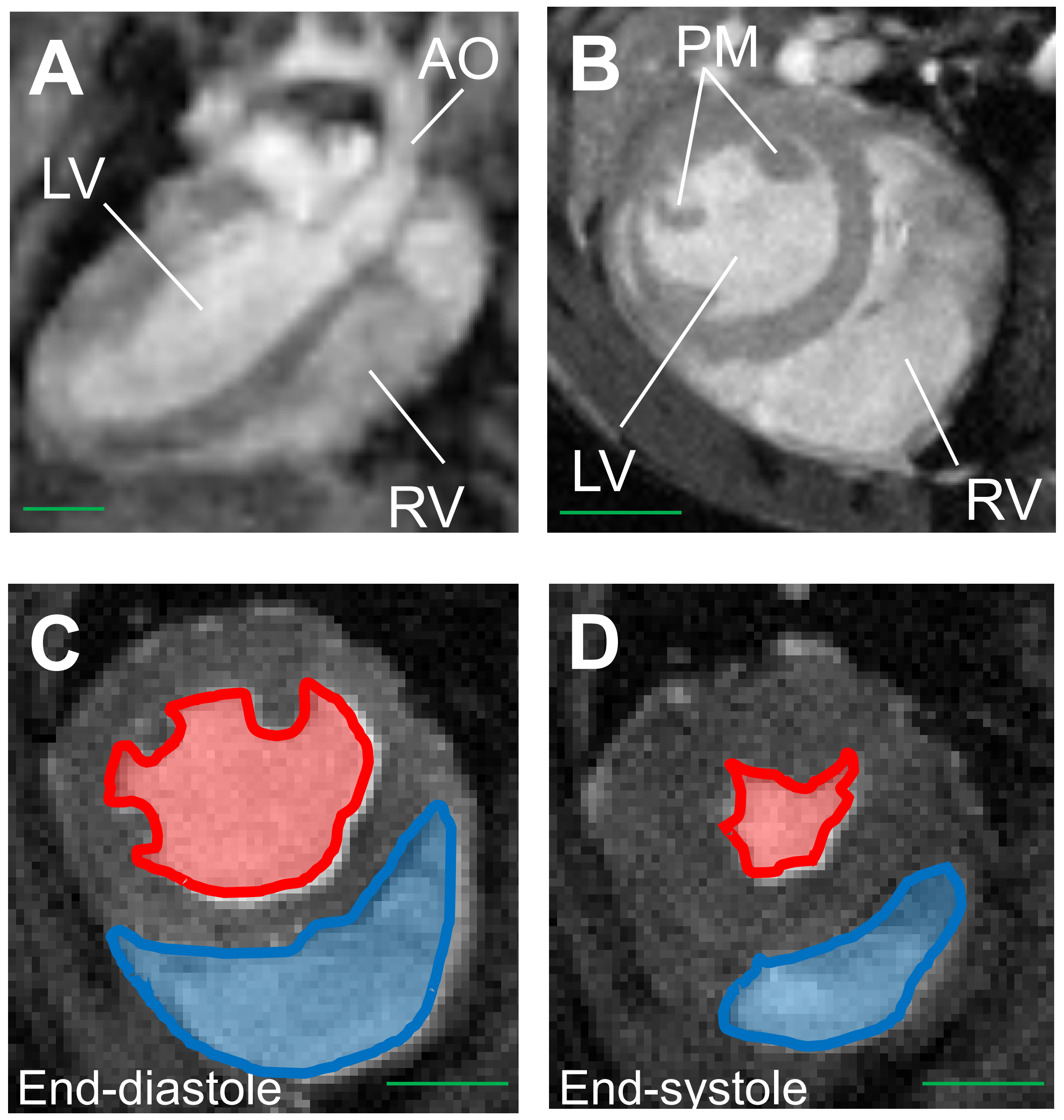

Supplementary Figure S1. Representative long and short axis cardiac MRI images of a mouse heart. **A.** End-diastole MR image in longitudinal orientation orthogonal to the interventricular septum. **B.** End-diastolic short-axis images at mid-ventricular level. Detailed cardiac compartments is indicated (Bar = 2 mm). **C, D.** Tracing short-axis MR images for end-diastole (C) and end-systole (D) volume determination of the left (red) and right (blue) ventricle. AO; aorta, LV, left ventricle; PM, papillary muscle, RV; right ventricle

Supplementary Figure S2

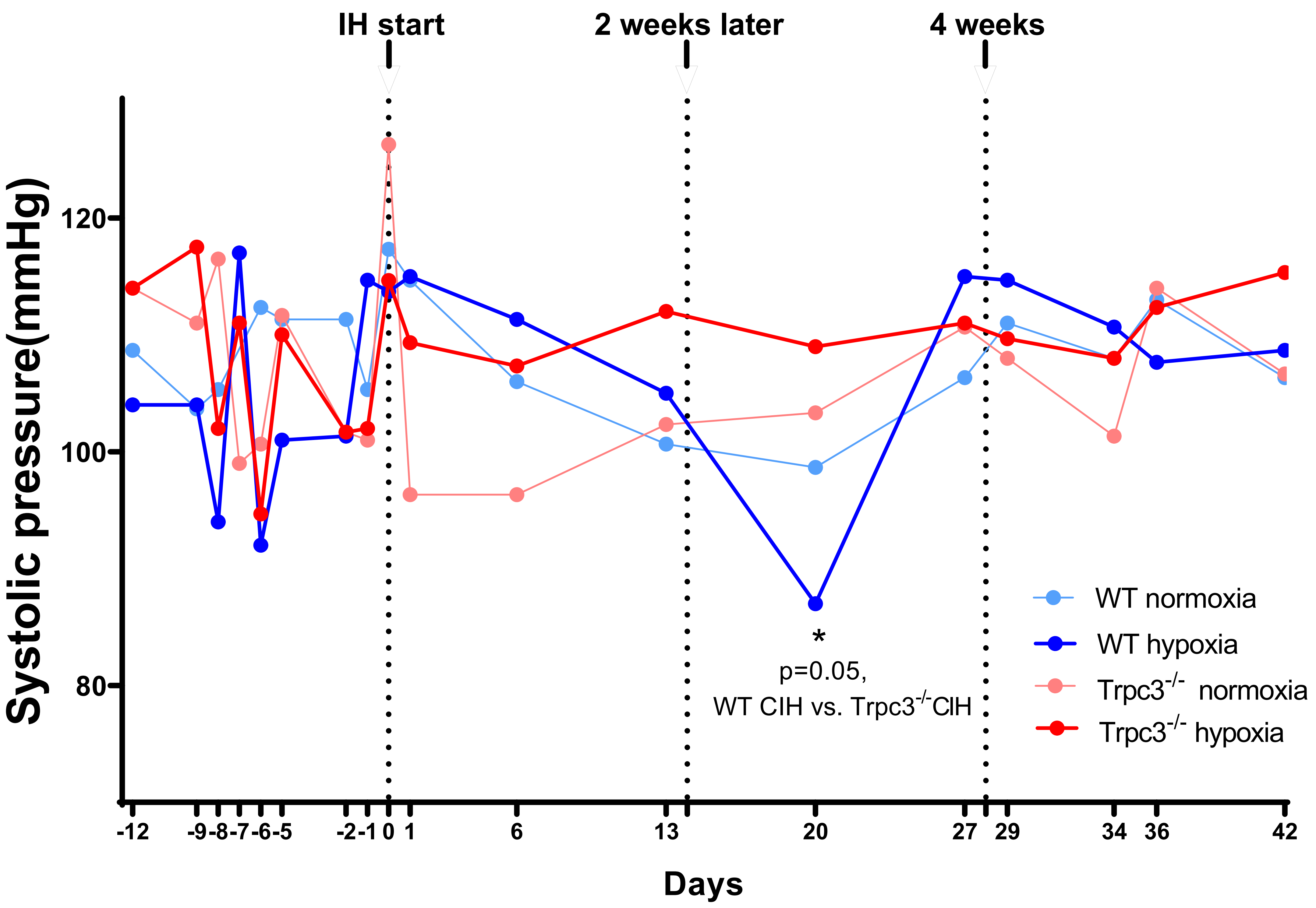

Supplementary Figure S2. Systolic blood pressure (BP) of WT and Trpc3$^{-/-}$ mice for 7 weeks of CIH condition. To analyze the effect of Trpc3 deletion on the CIH induced cardiovascular changes, we measured BP 2 weeks before chamber implement and 7 week after CIH condition. The BP was not significantly changed after CIH condition, except the BP of WT mice were significantly dropped at 3 weeks of CIH condition. Mouse BP was measured with noninvasive equipment, the two-chamber model of the BP-2000 BP analysis system (Visitech, Apex, NC, USA). Mice were adapted to caging in the measuring chamber and trained for tail cuff manipulation for 2 weeks before initiation of experiments. We monitored BP for approximately 2 weeks before treated in CIH chamber and 7 weeks of CIH treatment. The stage platforms were heated to 37°C. Ten primary BP measurements were taken, and 25 actual measurements were recorded. A minimum of five successful daily measurements were used in subsequent analyses.

Supplementary Figure S3

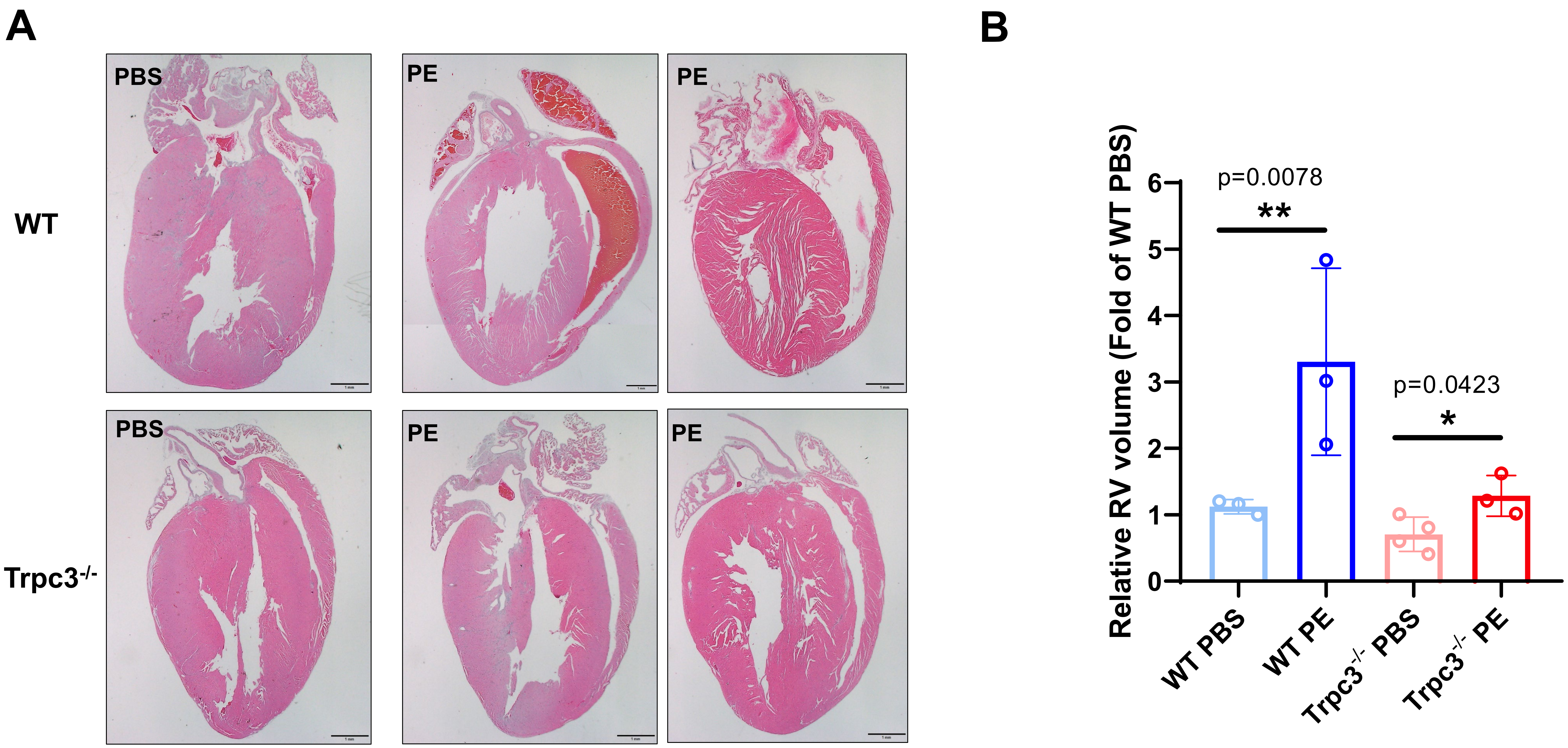

Supplementary Figure S3. A. The histology observed after H&E staining of WT and Trpc3$^{-/-}$ mice hearts after 4 weeks of PE implantation. **B.** Resistance of RV dilation of Trpc3$^{-/-}$ mice. Relative RV volume of WT and Trpc3$^{-/-}$ mice treated with PBS or PE were analyzed. Data are expressed as mean ± SE (n=3).
